# Activation of MAPK signalling results in resistance to saracatinib (AZD0530) in ovarian cancer

**DOI:** 10.18632/oncotarget.23524

**Published:** 2017-12-20

**Authors:** Niamh McGivern, Aya El-Helali, Paul Mullan, Iain A. McNeish, D. Paul Harkin, Richard D. Kennedy, Nuala McCabe

**Affiliations:** ^1^ Centre for Cancer Research and Cell Biology, Queen's University Belfast, Northern Ireland, UK; ^2^ Institute of Cancer Sciences, University of Glasgow, Scotland, UK; ^3^ Almac Diagnostics, 19 Seagoe Industrial Estate, Craigavon, Northern Ireland, UK

**Keywords:** ovarian cancer, SRC, MAPK, resistance, drug combination

## Abstract

SRC tyrosine kinase is frequently overexpressed and activated in late-stage, poor prognosis ovarian tumours, and preclinical studies have supported the use of targeted SRC inhibitors in the treatment of this disease. The SAPPROC trial investigated the addition of the SRC inhibitor saracatinib (AZD0530) to weekly paclitaxel for the treatment of platinum resistant ovarian cancer; however, this drug combination did not provide any benefit to progression free survival (PFS) of women with platinum resistant disease. In this study we aimed to identify mechanisms of resistance to SRC inhibitors in ovarian cancer cells. Using two complementary strategies; a targeted tumour suppressor gene siRNA screen, and a phospho-receptor tyrosine kinase array, we demonstrate that activation of MAPK signalling, via a reduction in NF1 (neurofibromin) expression or overexpression of HER2 and the insulin receptor, can drive resistance to AZD0530. Knockdown of NF1 in two ovarian cancer cell lines resulted in resistance to AZD0530, and was accompanied with activated MEK and ERK signalling. We also show that silencing of HER2 and the insulin receptor can partially resensitize AZD0530 resistant cells, which was associated with decreased phosphorylation of MEK and ERK. Furthermore, we demonstrate a synergistic effect of combining SRC and MEK inhibitors in both AZD0530 sensitive and resistant cells, and that MEK inhibition is sufficient to completely resensitize AZD0530 resistant cells. This work provides a preclinical rationale for the combination of SRC and MEK inhibitors in the treatment of ovarian cancer, and also highlights the need for biomarker driven patient selection for clinical trials.

## INTRODUCTION

Epithelial ovarian cancer (EOC) is the fifth most common cancer affecting women in the UK, and due to a lack of indicative symptoms, the majority of women will be diagnosed in the late stages (III-IV) of disease [1]. This late diagnosis results in poor prognosis and high mortality rates, thus this disease has been dubbed the silent killer [2, 3], with less than 35% of women surviving their disease for more than ten years [4] Survival rates have only marginally improved over the last 40 years, largely due to the heterogeneous biology that underpins the development of this disease, as well as the poor ability to detect the presence of EOC in early stages. Although many targeted therapies have been investigated in the treatment of EOC, the majority have failed to show any clear clinical benefit and upfront treatment remains debulking surgery followed by a platinum and taxane based chemotherapy regime. Following initial chemotherapy, the majority will relapse and eventually succumb to platinum/taxane resistant disease [5].

SRC is a non-receptor tyrosine kinase which has proven to be an important oncogene in various cancer types including breast [6], colorectal [7–9] and EOC [10, 11]. SRC has been reported to be overexpressed and activated in late stage poor outlook EOC [10] and *in vivo* xenograft data has shown that inhibition of SRC activity reduces tumour growth [11]. SRC activity has also been implicated in resistance of ovarian cancer cells to anti-estrogen therapies, and a combination of the SRC inhibitor saracatinib (AZD0530) and fluvestrant resulted in increased cell cycle arrest and decreased survival of ovarian cancer cells [12]. Furthermore, SRC has also been identified as a potential driver of resistance to paclitaxel in ovarian cancer cells, and SRC inhibition enhances the antitumour and antiangiogenic effects of paclitaxel [13–15]. These findings have supported the use of SRC inhibitors for the treatment of ovarian cancer in the clinic, and a number of phase I trials have shown the efficacy of SRC inhibitors to reduce phosphorylation of SRC (Tyr416) in a safe and tolerable manner in combination with platinum and taxane chemotherapy [16, 17]. In light of these findings, saracatinib (AZD0530), a potent kinase inhibitor with selective action against SRC was studied in combination with weekly paclitaxel in the phase II SAPPROC trial (NCT01196741) for women with recurrent platinum resistant EOC [18]. Surprisingly this study reported that the addition of AZD0530 to weekly paclitaxel did not improve progression free survival (PFS) [18].

Multiple studies have identified a number of mechanisms of resistance to inhibitors of the SRC pathway including activation of the mTOR pathway [19], suppression of autophagy [20] and secondary mutations in *DDR*, as well as loss of the tumour suppressor gene *NF1* [21]. It has also been reported that *PTTG1* expression is predictive of sensitivity in ovarian cancer cell lines to SRC inhibition with saractinib (AZD0530) [22]. However this work has not been performed in ovarian cancer models of acquired resistance to SRC inhibitors. We aimed to identify potential mechanisms of resistance to the SRC inhibitor AZD0530 in EOC by using two complementary screening methods and novel models of acquired resistance to AZD0530, and identified MAPK signalling as a potential predictive biomarker for SRC inhibitor resistance and for combination drug therapy.

## RESULTS

### A targeted tumour suppressor gene siRNA screen identifies loss of *NF1* as a mediator of AZD0530 resistance

**Figure 1:**
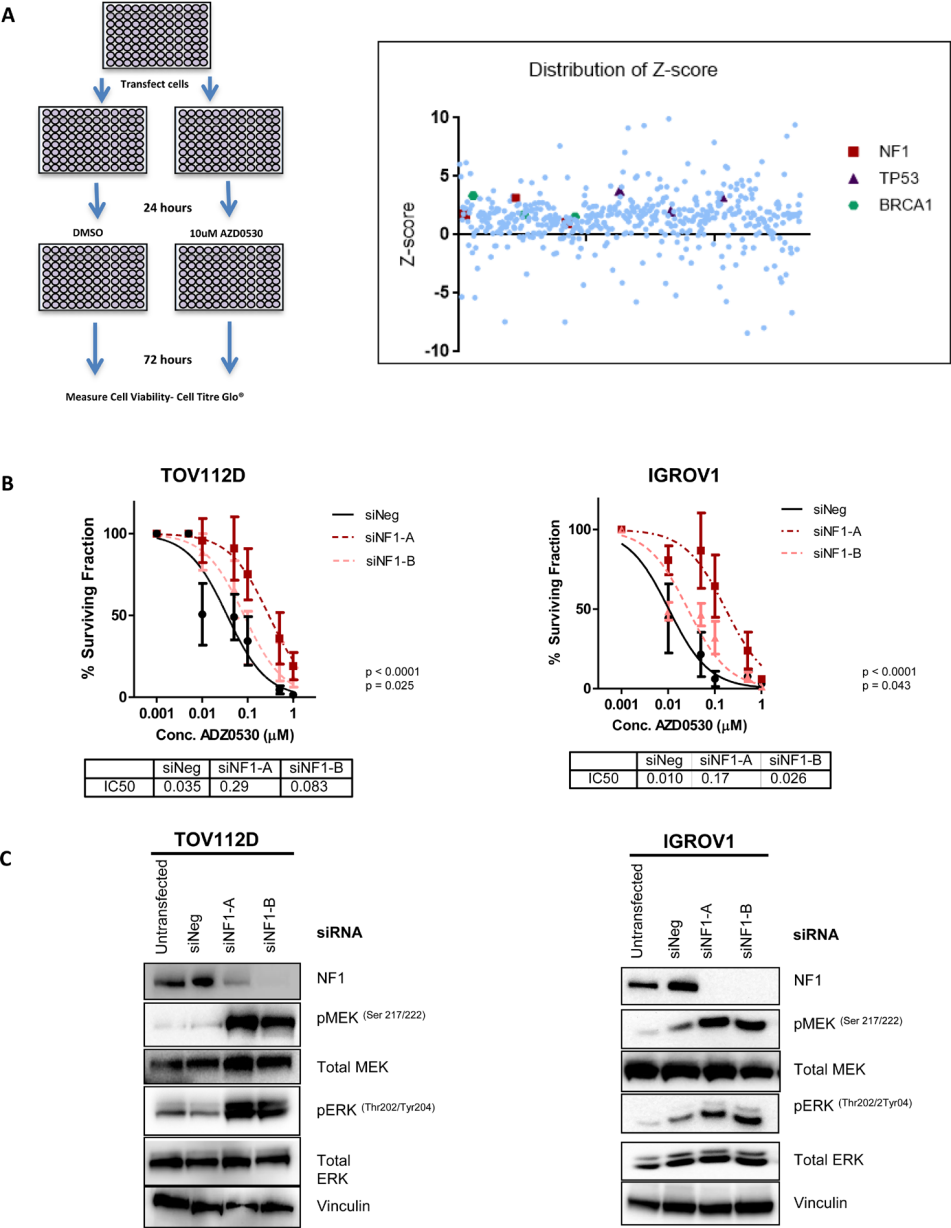
A targeted tumour suppressor gene siRNA screen identifies loss of NF1 as a mechanism of resistance to SRC inhibition (**A**) siRNA screening method. HFF cells were reverse transfected with 3 independent siRNAs targeting 178 tumour suppressor genes and 24 hours later were treated with either DMSO control or 10 uM AZD0530. Cell viability was recorded 72 hours later. Scatter plot showing distribution of robust z-scores for tumour suppressor gene siRNA screen. Positive scores indicate potential mediators of resistance to AZD0530, while negative scores indicate potential mediators of sensitivity to AZD0530. Each siRNA targeting NF1 is highlighted in red. Pathway analysis and gene ontology identified a list of genes which were involved in the regulation of protein kinase activity. (**B**) Colony formation assay assessing the sensitivity of TOV112D and IGROV1 cells to increasing concentrations of AZD0530 following transfection with negative control siRNA, or two independent siRNAs targeting NF1. IC50 values are shown. (**C**) Representative western blot analysis showing levels of NF1, phospho-MEK (Ser217/222), phospho-ERK (Thr202/Tyr204) following transfection of TOV112D and IGROV1 with negative control siRNA, and two independent siRNAs targeting NF1. Levels of total MEK and ERK are also shown, and vinculin levels are shown as a loading control. Western blotting analysis is representative of at least 3 independent biological replicates. *P* values are shown and were calculated using extra-sum of squares *F*-test. Data represented as mean ± S.E.M *n* ≥ 3.

A customized siRNA library targeting 178 tumour suppressor genes (TSG) (Supplementary Table 1) was used to identify those tumour suppressors whose knock-down confers resistance to AZD0530. Human foreskin fibroblast (HFF) cells were used for screening purposes as they are less likely to contain any pre-existing alterations in TSGs [23]. An IC50 for AZD0530 in these cells was determined as 10 μM, which resulted in a reduction in the levels of phosphorylated FAK (Supplementary Figure 1A), a downstream target of SRC kinase activity. Following transfection of HFF cells with the siRNA library, and treatment with either DMSO or 10 μM AZD0530, cell viability was measured 72 hours later (Figure 1A). Target genes were defined as resistant hits when each of the 3 independent siRNAs had a robust z-score greater or less than ±1 respectively. We identified 53 resistant hits (Supplementary Table 2). To select potential hits which are relevant to ovarian cancer, we cross- referenced the list of resistant hits with the most frequently occurring mutations in high-grade serous ovarian cancer (HGSOC) [24]. We identified that knockdown of *NF1, BRCA1* and *TP53* lead to decreased sensitivity to AZD0530, and were one of the most frequently mutated genes in HGSOC. To validate the findings from the library screen we independently knocked down *NF1, BRCA1* and *TP53* in HFF cells using an alternative siRNA sequence and investigated sensitivity to AZD0530 by cell count after 10 days (Supplementary Figure 1B). RNAi mediated knockdown of *BRCA1* and *TP53* did not result in loss of sensitivity to AZD0530 (Supplementary Figure 1B), while, *NF1* knockdown resulted in decreased sensitivity to AZD0530 compared to a negative control siRNA, with an increase in IC50 from 0.16 μM to 0.35 μM (fold change 2.2) (Supplementary Figure 1B). To further investigate whether loss of BRCA1 or P53 expression resulted in decreased sensitivity to AZD0530, we tested isogenic cell lines MDA-MB-436-E.V (empty vector) and matched MDA-MB-436 +BRCA1 [25], and MCF10A parental cells and matched MCF10A P53–/– [26]. Colony formation assay showed that the BRCA1 mutant MDA-MB-436-E.V cell line was in fact more sensitive to AZD0530 than MDA-MB-436 + BRCA1 cells further demonstrating that loss of BRCA1 does not result in resistance to SRC inhibition (Supplementary Figure 2C). Furthermore, colony formation assay showed that MCF10A breast cells which have *TP53* knockout, did not exhibit decreased sensitivity to SRC inhibition compared to parental MCF10A P53 WT cells. NF1 was therefore selected for further investigation in two ovarian cancer cell lines.

To select the most appropriate cell line models, we tested the sensitivity of a panel of ovarian cancer cells to AZD0530 by colony formation assay. TOV112D and IGROV1 EOC cells displayed high sensitivity to SRC inhibition with AZD0530, with IC50s of 230 nM and 370 nM respectively, indicating a reliance on SRC signaling for survival and growth (Supplementary Figure 2). *NF1* was silenced in both TOV112D and IGROV1 cells using two independent siRNAs, and sensitivity to AZD0530 assessed by a colony formation assay. Consistent with a role in resistance to AZD0530, knockdown of *NF1* with both siRNAs resulted in a significant increase in the IC50 for both TOV112D cells, from 0.035 μM to 0.29 μM (*p <* 0.0001) and 0.083 μM (*p* = 0.043) (fold change 8.29, 2.37 respectively) and IGROV1 cells, from 0.01 μM to 0.17 μM (*p <* 0.0001) and 0.026 μM (*p* = 0.028) (fold change 16.5, 2.56 respectively) (Figure 1B). The protein product of *NF1,* neurofibromin, inhibits mitogen-activated protein kinase (MAPK) dependent proliferation and differentiation pathways [27, 28]. As expected, siRNA mediated knockdown of *NF1* using two siRNAs in TOV112D and IGROV1 cells led to increased MEK and ERK phosphorylation in both TOV112D and IGROV1 cells (Figure 1C), indicating MAPK activation.

### Saracatinib (AZD0530) resistant ovarian cell lines exhibit increased HER2 and insulin receptor expression

**Figure 2:**
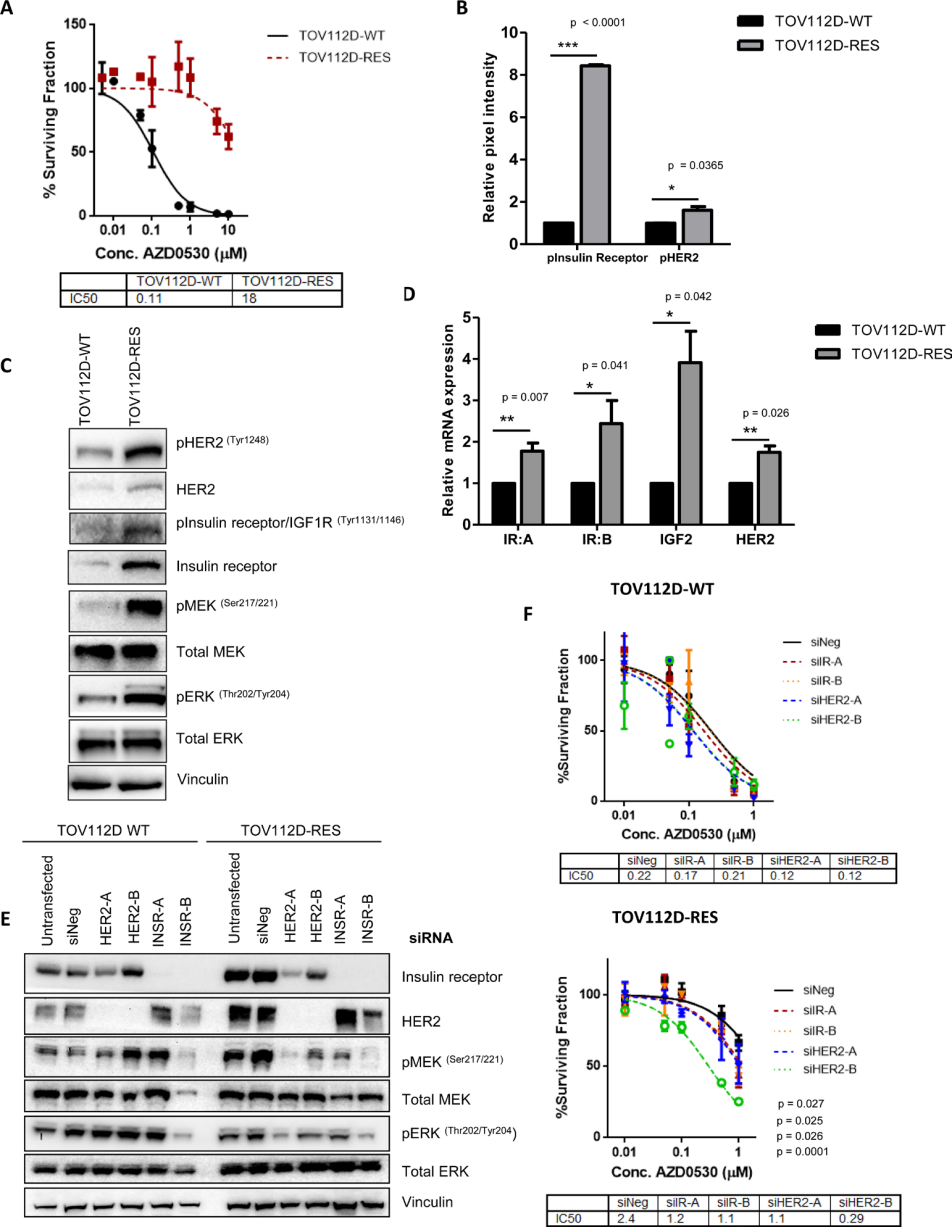
A phospho-RTK array identifies activation of HER2 and insulin receptor as mediators of resistance to AZD0530 (**A**) 10 day colony formation assay assessing sensitivity of TOV112D-WT and TOV112D-RES cells to increasing concentrations of AZD0530. IC50 values are shown. (**B**) Relative pixel intensity of levels of phospho-HER2 and phospho-Insulin receptor in phospho-RTK array (**C**) Representative western blot analysis showing levels of phosphorylated and total HER2 protein expression, phosphorylated insulin/IGF1R and total levels of the insulin receptor, and phosphorylated MEK (Ser217/221) and ERK (Thr202/Tyr204) in TOV112D WT and TOV112D-RES cells. Total MEK and ERK levels are also shown. Vinculin was used as a loading control. (**D**) Quantitative real-time polymerase chain reaction (qRT-PCR) analysis of relative mRNA expression of insulin receptor isoform A (IR:A), IGF-2, insulin receptor isoform B (IR:B) and HER in TOV112D-WT and TOV112D-RES cells. (**E**) Representative western blot analysis showing levels of total HER2 and insulin receptor protein expression following transient transfection of TOV112D-WT and TOV112D-RES cells with two siRNAs targeting the insulin receptor and HER2. Levels of phosphorylated MEK (Ser217/221) and ERK (Thr202/204) are shown, as well as total levels of MEK and ERK. Vinculin was used as a loading control. (**F**) Colony formation assay showing sensitivity of TOV112D-WT and TOV112D-RES cells to increasing concentrations of AZD0530 following treatment with two siRNAs targeting the insulin receptor or the HER2 receptor. IC50 values are shown. *P* values are shown and were calculated using extra-sum of squares *F*-test for drug sensitivity assays. Western blotting analysis is representative of at least 3 independent biological replicates. Statistical significance comparing phospho-RTK and gene expression data was calculated using an unpaired students *t*-test. ^*^ = *p* < 0.05 ^**^= *p* < 0.01 ^***^p < 0.001. Data represented as mean ± S.E.M *n* ≥ 3.

To complement the siRNA screen, we used a further strategy to investigate mechanisms of resistance to AZD0530. To do this we generated AZD0530 resistant EOC cell lines *in vitro* and used these for screening purposes. TOV112D cells were cultured in AZD0530 for 6 months after which resistance was confirmed by a significant increase in colony formation assay following treatment with AZD0530 (Figure 2A). TOV112D resistant cells (TOV112D-RES), exhibited a 165 fold increase in their IC50 for AZD0530 compared to the parental cell line (TOV112D-WT), from 0.11 μM to 18 μM (*p <* 0.0001) (Figure 2A). These resistant cells were also shown to have a significant increase in survival by colony formation assay following RNAi mediated SRC knockdown compared to TOV112D-WT cells (*p* = 0.0055, *p* = 0.019) (Supplementary Figure 3A), thus demonstrating no requirement for SRC activity. Next we asked if AZD0530 was still able to inhibit SRC activity in these resistant cells. Following treatment with AZD0530 there was a decrease in levels of phosphorylated SRC (Tyr416) and FAK (Tyr925) in both TOV112D-WT and TOV112D-RES cells, indicating that the drug was on-target (Supplementary Figure 3B). Interestingly, we also observed a decrease in the levels of total SRC protein expression in TOV112D-RES cells compared to TOV112D-WT cells (Supplementary Figure 3A and 3B). Combined with the fact that AZD0530 was still preventing phosphorylation of FAK Y295, this indicates that TOV112D-RES cells no longer require SRC activity or expression for survival and growth, and activation of alternative survival pathways may therefore be driving drug resistance.

A second AZD0530 resistant ovarian cell line was generated by culturing IGROV1 cells in AZD0530 (IGROV1-RES). IGROV1-RES cells exhibited a 5 fold increase in their IC50 for AZD0530 compared to their parental cell line (IGROV1-WT) from 0.087 μM to 0.44 μM (*p <* 0.0001) (Supplementary Figure 3D) and similarly treatment of both IGROV1-WT and IGROV1-RES cells with AZD0530 resulted in a decrease in the phosphorylation of both SRC (Tyr416) and FAK (Tyr925) (Supplementary Figure 3E).

Since receptor tyrosine kinases (RTKs) are important mediators of downstream signaling pathways and have also been implicated in resistance to both conventional chemotherapies as well as targeted agents [29–32], we investigated whether RTK expression was associated with saracatinib resistance. We used TOV112D-WT and TOV112D-RES cells to screen a panel of 49 RTKs and identified a significant increase in the activation of the HER2 receptor and insulin receptor in the AZD0530 resistant cells (Figure 2B, Supplementary Figure 3C). These findings were confirmed by western blotting, whereby TOV112D-RES cells exhibited increased phosphorylation and expression of both HER2 and the insulin receptor, and was accompanied with increased phosphorylation of MEK and ERK, indicating activation of MAPK signaling, compared to TOV112D-WT cells (Figure 2C). Similarly, an increase in expression and phosphorylation of HER2 and the insulin receptor accompanied by activated MEK and ERK signaling was also demonstrated in IGROV1-RES cells compared to IGROV1-WT (Supplementary Figure 3F). The insulin receptor is known to exist as two splice variants, insulin receptor-A (IR:A) and insulin receptor-B (IR:B) [33], where IR:A is preferentially activated by IGF-II over insulin, and signals via the MAPK pathway to induce mitogenic signaling. We observed a significant increase in the mRNA expression of both IR:A (*p* = 0.007) and IR:B (*p* = 0.041) as well as a significant increase in the mRNA levels of the IR:A ligand IGF-II (*p* = 0.042) in the TOV112D-RES cells compared to TOV112D-WT cells (Figure 2D). In addition we observed a significant increase in the expression of HER2 mRNA (*p* = 0.0026) in TOV112D-RES cells compared to TOV112D-WT.

To investigate if the observed increase in MAPK activation was a direct result of increased expression and activation of each of these receptors, we silenced each receptor using two independent siRNAs in TOV112D-WT and TOV112D-RES cells. Knockdown of each of these receptors resulted in a decrease in MEK and ERK phosphorylation in TOV112D-RES, but not in TOV112D-WT cells, suggesting that the observed increase in MAPK activation is a direct consequence of increased HER2 and insulin receptor expression (Figure 2E). Interestingly, we also observed that siRNA mediated knockdown of HER2 resulted in decreased expression of insulin receptor levels specifically in TOV112D-RES cells, but not in TOV112D-WT cells. This indicates that HER2 may be driving the observed increased expression of the insulin receptor in TOV112D-RES cells, and so both of these receptors may be working together to drive activation of MAPK signaling, and therefore resistance to SRC inhibition (Figure 2E). Cooperation of HER2 and the insulin/IGF1R pathway has been previously implicated in resistance to targeted therapies, whereby in a model of trastuzumanb resistant breast cancer, HER2 and IGF1R form heterodimers, and stimulation of IGF1R leads to HER2 phosphorylation in resistant cells, but not in sensitive cells [34].

To confirm that the increased expression and activation of HER2 and the insulin receptor could account for resistance to SRC inhibition, we tested the colony formation ability of each cell line in the presence of AZD0530 following silencing of both receptors using two siRNAs. Silencing of HER2 and the insulin receptor did not result in increased sensitivity of TOV112D-WT cells to AZD0530, however, silencing of both of these receptors increased sensitivity of TOV112D-RES cells to AZD0530, compared to negative control transfected cells. This led to a decrease in the IC50 of TOV112D-RES cells from 2.39 μM to 1.180 μM (*p* = 0.027), 1.13 μM (*p* = 0.025), 1.037 μM (*p* = 0.026) and 0.29 μM (*p* < 0.0001) following transfection with a negative control siRNA, and two siRNAs targeting the insulin receptor and HER2, respectively (Figure 2F). Notably, silencing of either HER2 or the insulin receptor alone resulted in a partial rescue in sensitivity of TOV112D-RES cells to AZD0530, suggesting that these receptors act in parallel to confer resistance.

### SRC and MEK act in parallel pathways

**Figure 3:**
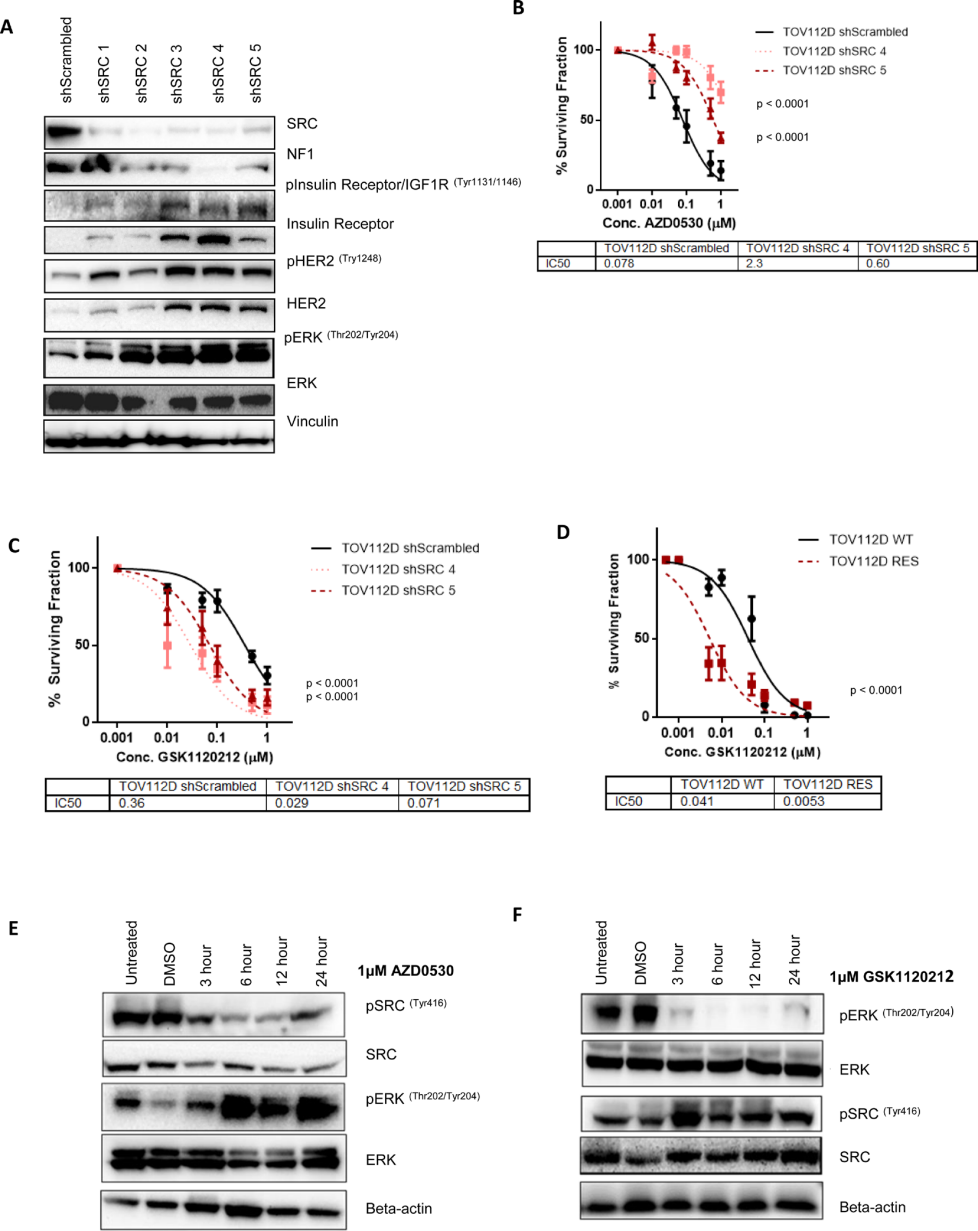
SRC and MEK act as parallel and compensatory pathways (**A**) Representative western blot analysis of 5 stable SRC knockdown clonal cell populations and a scrambled shRNA control cell population (shScrambled) generated in TOV112D cells. NF1, phospho-insulin receptor/IGF1R, phopsho-HER2 and phospho-ERK levels are shown. Total levels of the insulin receptor, HER2 and ERK are also shown. Vinculin expression was used as a loading control. (**B**) 10 day colony formation assay showing sensitivity of TOV112D shScrambled, TOV112D shSRC 4 and TOV112D shSRC 5 to increasing concentrations of AZD0530. IC50 values are shown (**C**) 10 day colony formation assay showing sensitivity of TOV112D shScrambled, TOV112D shSRC 4 and TOV112D shSRC 5 to increasing concentrations of trametinib (GSK1120210) IC50 values are shown. (**D**) 10 day colony formation assay assessing sensitivity of TOV112D-WT and TOV112D-RES cells to increasing concentrations of GSK1120212. IC50 values are shown. (**E**) Representative western blot analysis showing levels of ERK and SRC phosphorylation following treatment of TOV112D cells with either 1 μM saracatainib (AZD0530) for 3, 6, 12, and 24 hours. Total levels of SRC and ERK are also shown. Beta-actin expression was used as a loading control. (**F**) Representative western blot analysis showing levels of ERK and SRC phosphorylation following treatment of TOV112D cells with 1μM trametinib (GSK1120212) for 3, 6, 12, and 24 hours. Total levels of SRC and ERK are also shown. Beta-actin expression was used as a loading control. Western blotting analysis is representative of at least 3 independent biological replicates. *P* values are shown and were calculated using extra-sum of squares *F*-test. Data presented represents mean ± S.E.M *n* ≥ 3.

To further validate our findings from the TSG siRNA screen and the phospho-RTK array, we generated stable SRC knockdown cell lines to represent a cell population that can survive in the absence of SRC signaling, thereby modeling resistance to SRC inhibition. We transfected TOV112D cells with a SRC targeted shRNA, and isolated clones. We examined the expression of NF1, the insulin receptor and HER2 in the stable SRC knockdown cells. Interestingly, we found that 5 of the stable SRC knockdown clones demonstrated decreased expression of the *NF1* gene product neurofibromin. Furthermore, each of these clones also exhibited increased phosphorylation and expression of both the HER2 and insulin receptor (Figure 3A). Each of the SRC knockdown clones exhibited increased MAPK signaling, with increased levels of ERK phosphorylation compared to the shScrambled control cell lines (Figure 3A), supporting its role in promoting cellular survival in the absence of SRC signaling.

To confirm that stable knockdown of SRC was in fact a model of resistance to SRC inhibition, we tested sensitivity of two SRC knockdown clones to AZD0530. We selected TOV112D-shSRC4 and TOV112D-shSRC5 as they demonstrated the most prominent knockdown of SRC, loss of NF1 expression, and the biggest increase in insulin receptor and HER2 activation and expression. We found that TOV112D-shSRC4 and TOV112D-shSRC5 exhibited decreased sensitivity to SRC inhibition with AZD0530 by colony formation, with an increase in IC50 from 0.078 μM in the shScrambled cells to 2.3 μM (*p* < 0.0001) for shSRC4 and 0.6 μM for shSRC5 (*p* < 0.0001) (Figure 3B).

In order to confirm the requirement for MAPK signaling in the SRC knockdown cell lines, we tested sensitivity to the MEK inhibitor trametinib. Consistent with a role for MAPK signaling in promoting survival, two SRC knockdown clones exhibited increased sensitivity to MEK inhibition with a decrease in the IC50 from 0.36 μM in the shScrambled cells to 0.0029 μM for shSRC 4 (*p <* 0.0001) and 0.071 μM for shSRC 5 (*p <* 0.0001), resulting in a fold change in the IC50 of 12.2 and 5 respectively (Figure 3C).

Given the observed increased sensitivity of the stable SRC knockdown clones to MEK inhibition with trametinib, we then tested the requirement for MEK activity for the survival of the AZD0530 resistant TOV112D-RES cell line. Colony formation assay demonstrated that TOV112D-RES cells were significantly more sensitive to MEK inhibition compared to TOV112D-WT cells, with a decrease in the IC50, from 0.041 μM to 0.0053 μM (*p <* 0.0001) respectively (fold change 7.8), suggesting a reliance on MEK signalling in the AZD0530 resistant line (Figure 3D).

We then investigated the acute effect of SRC inhibition with AZD0530 on MEK signalling. Following treatment of TOV112D cells with 1 μM AZD0530 over a 24 hour time-course, we observed decreased phosphorylation of SRC (Tyr416). Interestingly, this coincided with an increase in phosphorylation of ERK (Th202/Tyr204) again supporting parallel functions of the SRC and MAPK pathways (Figure 3E). We then asked if this was a compensatory relationship, and inhibited MEK in TOV112D cells with GSK1120212 over the same time course. We observed a decrease in ERK phosphorylation (Th202/Tyr204) and a concomitant increase in SRC phosphorylation (Tyr416) (Figure 3E). Together these data suggest that the SRC and MAPK pathway are parallel and compensatory, and inhibition of one leads to activation of the other, allowing for cell survival and proliferation, resulting in drug resistance.

### Dual inhibition of saracatinib (AZD0530) and trametinib (GSK1120212) synergistically inhibits colony formation in saracatinib resistant cells

**Figure 4:**
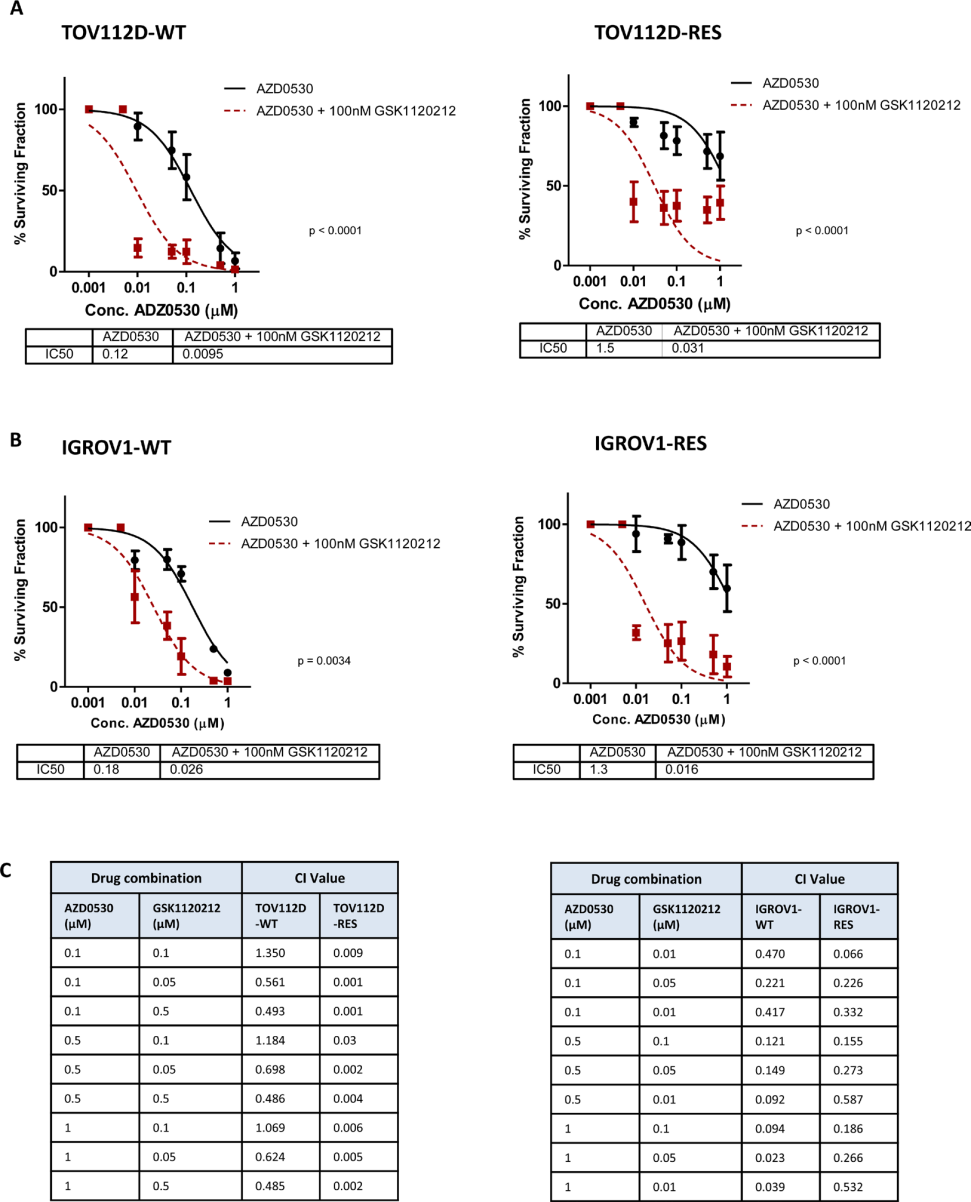
Dual inhibition of MEK and SRC synergistically inhibits growth of ovarian cancer cells, and MEK inhibition can resensitize AZD0530 resistant cells (**A**) 10 day colony formation assay showing sensitivity of TOV112D-WT and TOV112D-RES to increasing concentrations to increasing concentrations of AZD0530 as single agent or in combination with 100nM trametinib (GSK1120212). IC50 values are shown. (**B**) 10 day colony formation assay showing sensitivity of IGROV1-WT and IGROV1-RES to increasing concentrations to increasing concentrations of AZD0530 as single agent or in combination with 100nM trametinib (GSK1120212). IC50 values are shown. (**C**) Combination index (CI) values for TOV112D-WT and TOV112D-RES, and IGROV1-WT and IGROV1-RES cells following treatment with various doses of a combination of saracatinib (AZD0530) and trametinib (GSK1120212). CI value <1 indicates drug synergy. *P* values are shown and were calculated using extra-sum of squares *F*-test. Data represented as mean ± S.E.M *n* ≥ 3.

To investigate the therapeutic potential of combining SRC and MEK inhibitors we treated both the TOV112D and IGROV1 cells, and their respective AZD0530 resistant counterparts with increasing concentrations of AZD0530, either as a single agent, or in combination with the 100nM MEK inhibitor GSK1120212. Colony formation was assessed after a 10 day period, and for combination treatments, survival was calculated as a percentage of the effect of 100 nM GSK1120212, to demonstrate any synergistic effects. The combination of GSK1120212 and AZD0530 resulted in a significant decrease in colony formation of both TOV112D-WT and TOV112D-RES cells compared to AZD0530 treatment alone. This resulted in a decrease in IC50 from 0.1247 μM to 0.0095 μM (*p* < 0.0001 fold change 26) for TOV112D-WT cells, and a decrease in IC50 from 1.503 μM to 0.0306 μM (*p <* 0.0001) (fold change 49) for TOV112D-RES cells (Figure 4A). Similarly, the combination of GSK1120212 with AZD0530 resulted in a significant decrease in survival of the second *in vitro* derived AZD0530 resistant cell line, IGROV1-RES, and its matched parental cell line, IGROV1-WT, compared to treatment with AZD0530 alone. This resulted in a decrease in the IC50 from 0.18 μM to 0.026 μM (*p* = 0.034 fold change 4.5) for IGROV1-WT cells, and a decrease in IC50 from 1.28 μM to 0.016 μM (fold change 80) for IGROV1-RES cells (*p* < 0.0001) (Figure 4B). The greater fold change in the IC50 of both AZD0530 resistant cell lines suggested a reliance on MAPK signalling for survival in the presence of SRC inhibition in TOV112D-RES and IGROV1-RES cells compared to their parental cell lines. To confirm a synergistic effect of the combination of MEK inhibition and SRC inhibition, combination index (CI) values were calculated (Figure 4C). The combination of AZD0530 and GSK1120212 was found to be synergistic in TOV112D-WT, TOV112D-RES cells, IGROV1-WT and IGROV1-RES cells. However, increased levels of drug synergy were observed in TOV112D-RES cells, (CI values ranging from 0.001 to 0.03) when compared to TOV112D-WT cells (CI values ranging from 0.486 to 1.350) treated with the same combination of AZD0530 and GSK1120212 indicating a greater reliance on MEK activation for survival of AZD0530 resistance cells in the presence of the inhibitor.

## DISCUSSION

The use of targeted therapies in the treatment of solid malignancies is often limited by the rapid development of drug resistant disease, hence there is a drive in the field of molecular oncology towards a better understanding of potential mechanisms of resistance to targeted small molecule inhibitors [35].

This phenomenon of rapid acquired or intrinsic resistance may explain the findings of the SAPPROC trial (NCT01196741), which investigated the use of the SRC inhibitor saracatinib (AZD0530) in combination with weekly paclitaxel, for the treatment of platinum resistant ovarian cancers. Despite promising preclinical studies which highlight the potential to target SRC in ovarian cancer, and provide a clear rationale for this clinical trial, results showed no benefit to progression free survival (PFS) when combining saracatinib (AZD0530) with weekly paclitaxel in this patient population [18]. We set out to investigate potential mechanisms of resistance to the SRC inhibitor saracatinib (AZD0530) in ovarian cancer, with the aim to highlight potential combination therapies and treatment stratification markers for SRC inhibitors in this disease.

**Figure 5:**
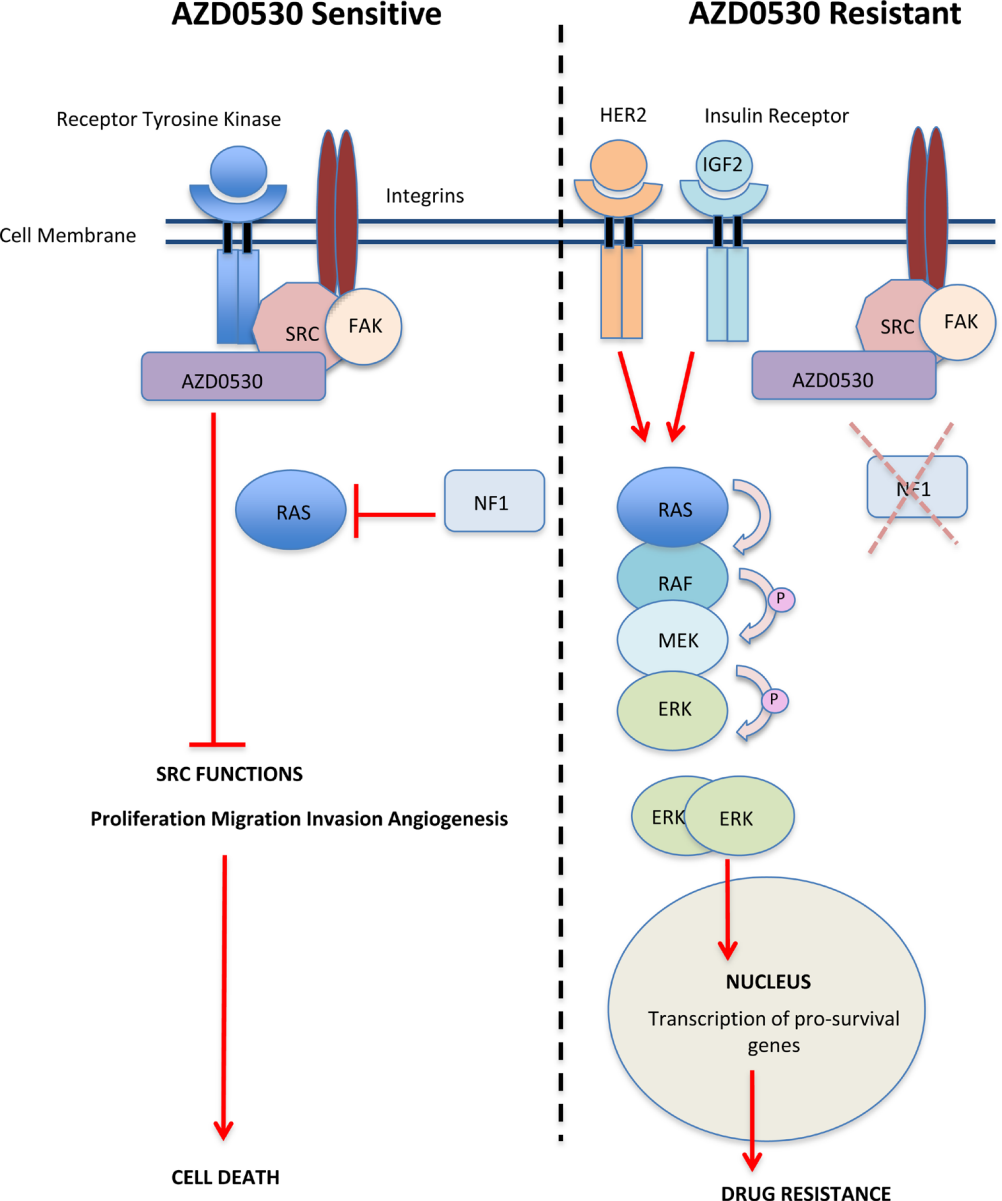
Schematic Model showing Activation of MAPK signalling Resulting in Resistance to AZD0530 **Left:** In saracatinib (AZD0530) sensitive cells, inhibition of SRC leads to a reduction in the ability of SRC to phosphorylate and activate downstream signalling pathways resulting in decrease proliferation, migration, invasion angiogenesis and cell death. **Right:** In saracatinib (AZD0530) resistant cells, increased expression and activation of HER2 and the insulin receptor drives the activation of the RAS-RAF-MAPK pathway. Loss or reduction in the expression of the tumour suppressor gene NF1 also releases the negative regulation of RAS-RAF MAPK pathways allowing for MAPK induced cell survival and drug resistance.

By using complementary screening approaches, a targeted tumour suppressor gene siRNA screen and a phopsho-RTK array, we have shown that loss of NF1 (neurofibromin) expression and increased activation and expression of both the HER2 receptor and the insulin receptor lead to resistance to saracatinib, via increased activation of the MAPK pathway (Figure 5).

Using a targeted tumour suppressor gene siRNA screen, we aimed to identify genes whose loss resulted in decreased sensitivity to AZD0530, hence the siRNA screen was designed to specifically identify mediators of resistance. To enrich for selection of mediators of resistance, we used an IC50 for AZD0530, and in doing so, we did not identify any genes whose knockdown resulted in increased sensitivity to AZD0530. The siRNA screen identified NF1 loss as a mediator of resistance to AZD0530. Loss of NF1 has been reported to result in resistance to various targeted tyrosine kinase inhibitors in a number of settings, including *BRAF*-mutant melanoma and *EGFR*-mutant lung cancer [36–38]. Consistent with our findings, mutation and subsequent loss of the tumour suppressor function of NF1 has been reported in lung cancer cells which have acquired resistance to the SRC inhibitor dasatinib, and this was associated with active RAS-MAPK signalling [21]. Here, we have demonstrated that reduction in the expression of NF1 in two ovarian cancer cell lines results in activated MEK and ERK signalling, and is associated with decreased sensitivity to SRC inhibition. Importantly, it has been reported that *NF1* is the fourth most altered gene in epithelial ovarian cancer [24], highlighting the relevance of this genetic alteration when targeting SRC in this disease setting.

By generating *in vitro* derived saracatinib (AZD0530) resistant ovarian cell lines we have demonstrated that overexpression and activation of both HER2 and the insulin receptor also results in resistance to SRC inhibition. Amplification of *ErbB2* (HER2) occurs in 28% of ovarian cancers [39], representing a potential therapeutic target in this subset of ovarian cancer patients. SRC activity has previously been linked to resistance to anti-HER2 therapies, with SRC activation correlating with trastuzumab resistance in breast cancer cells, and overexpression of an activated mutant form of SRC (Y527F) resulted in resistance to trastuzumab-mediated growth inhibition [40]. Furthermore, it has previously been shown that saracatinib resensitized gastroeosophageal cancer cell lines resistant to the HER2 inhibitor lapatinib [41].

We have also shown that knockdown of HER2 results in decreased levels of insulin receptor expression, specifically in TOV112D-RES cells, but not in TOV112D-WT cells.

We have also shown that saracatinib resistant cells express increased levels of both isoforms of the insulin receptor, IR:A and IR:B, as well as expressing increased levels of IGF2, the ligand for IR:A. Binding of IGF2 to IR:A has been reported to signal via the MAPK pathway, inducing a mitogenic response and driving cell cycle progression, mitosis and cell proliferation [42]. Interestingly, IGF signaling has previously been implicated in ovarian cancer progression and development, as well as resistance to targeted therapies and conventional chemotherapies [43–45]. Interestingly, increased SRC activity has been associated with resistance to anti-IGF-1R therapies. SRC and IGF-1R are reciprocally co-activated in high levels in non-small cell lung cancer (NSCLC) cells and inhibition of IGF-1R resulted in activation of SRC, and targeting SRC resensitzed cells to IGF-1R inhibition [46]. Similarly, a combination of anti-IGF-1R and SRC therapies displayed enhanced antitumour activity in *in vitro* and *in vivo* models of rhabdomyosarcoma when compared to either drug alone [47].

Our data demonstrates different mechanisms by which MAPK signalling can become activated and result in resistance to SRC inhibition in ovarian cancer cells, highlighting the potential for combining SRC inhibitors with small molecule inhibitors of the MAPK pathway. We have shown that a combination of SRC and MEK inhibition synergistically inhibits the growth of ovarian cancer cell lines, and that MEK inhibition is sufficient to resenesitize ovarian cancer cell lines with acquired resistance to saracatinib (AZD0530).

The efficacy of combining SRC and MEK inhibitors has been previously investigated in various cancer cell lines. In melanoma cancer cells, a combination of MEK and SRC inhibition suppressed a MEK inhibitor driven invasive phenotype associated with epithelial to mesenchymal transition (EMT) [48]. Moreover, melanoma cells made resistant to the BRAF inhibitor vemurafinib, which acts upstream of MEK activation and signalling, exhibited activated SRC signalling *in vitro* and *in vivo,* and exhibited sensitivity to SRC inhibition by both dasatinib and saracatinib [49]. Growth of vemurafinib resistant patient derived xenografts (PDX) was suppressed following treatment with the SRC inhibitor dasatinib, and reduced metastases [49]. A combination of SRC and MEK inhibitors has also proved beneficial in the treatment of breast cancer cells, where the combination of selumetinib and saracatinib induced apoptosis and reduced the initiation of both dormant and metastatic tumour cells, leading to diminished lung metasteses, compared to saracatinib alone [50]. Furthermore, in non-small cell lung cancer cell lines, a combination of the MEK inhibitor PD0325901 with saracatininb (AZD0530) abrogated tumour growth, enhanced mesenchymal to epithelial transition (EMT) and reduced cell migration and invasion [51]. Similar to our findings, recent work has also demonstrated the utility of combining SRC and MEK inhibitors in the ovarian cancer setting [52], where the combination of selumetinib and saracatinib abolished Rac-1 mediated EMT of ovarian cancer cells. Furthermore, this drug combination suppressed tumour progression more effectively than either drug alone and prolonged survival of mice transplanted with intraperitoneal xenografts [52].

Here we have shown that MAPK activation, via various mechanisms, leads to resistance to saracatinib. Interestingly, MAPK activation has also been associated with resistance to platinum based chemotherapies in ovarian cancer [53–55], and notably, patient recruitment to the SAPPROC clinical trial was based on resistance to platinum therapy [18]. This highlights the potential issues which led to failure of saracatinib to provide any benefit to PFS of women receiving weekly paclitaxel in the treatment of ovarian cancer in the SAPPROC trial. We have shown that a combination of SRC and MEK inhibitors synergistically inhibits the growth of saracatinib resistant ovarian cancer cells, and so we propose that ovarian cancer patients are more likely to respond to SRC inhibition in combination with MEK inhibition. Our findings, to our knowledge, are the first to highlight the potential of combining SRC and MEK inhibitors in the treatment of platinum resistant ovarian cancer.

## MATERIALS AND METHODS

### Generation of novel cell lines

To generate a cell line with stable shRNA mediated knockdown of SRC, TOV112D cells were infected with lentivirus particles containing SRC specific shRNA, and selected in puromycin (Supplementary Information). Saracatinib (AZD0530) resistant cells were generated by culturing TOV112D and IGROV1 cells in 5 μM AZD0530 for 6 months. Once resistance to AZD0530 was established, TOV112D-RES and IGROV1-RES cells were maintained in 1 μM AZD0530.

### siRNA screening

A customised siRNA library targeting 178 tumour suppressor genes or genes whose loss of function was associated with cancer (Supplementary Table 1) was acquired using predesigned siRNA sequences (Qiagen). The library contained 3 independent siRNA sequences per gene and was provided in an arrayed format in 96 well plates. Human foreskin fibroblasts (HFFs) cells were reverse transfected with the siRNA library (Qiagen) using Lipofectamine RNAiMax reagent (Life Technologies) (Supplementary Information). Cell viability was measured using Cell Titre Glo (Promega) 96 hours later. Results were median-centred, log transformed and normalised to scrambled siRNA negative control. Hits with robust z-scores of ±1 median absolute deviation (MAD) were carried forward for validation studies.

### Phospho-receptor tyrosine kinase array

Proteome Profiler Human Phospho-RTK Array Kit (RnD Systems) was performed according to manufacturer's guidelines. Briefly, cell lysates were prepared in RIPA buffer from TOV112D-WT and TOV112D-RES cells, 500μg of protein was incubated with each membrane. Densitometry analysis was performed using Image J (Fiji).

### Cell count assay

Human foreskin fibroblasts were reverse transfected with 10 nmol of gene specific siRNA (Qiagen) using Lipofectamine RNAiMax transfection reagent (Life Technologies), and 48 hours post transfection cells were trypsinized, counted and re-seeded into 6 well plates. 24 hours later, media was replaced with increasing concentrations of AZD0530. After 10 days, cells were trypsinized and counted. The % survival fraction for a given dose/siRNA was calculated and dose-response curves were plotted.

### Colony formation assays

Cells were seeded at predetermined densities, and 24 hours later treated with increasing concentrations of saracatinib (AZD0530) or trametinib (GSK1120212), which was replenished every 3-4 days. Where appropriate, cells were transfected with 10 nmol of gene-specific targeting siRNAs (Qiagen) using Lipofectamine RNAiMax transfection reagent (Life Technologies) and 48 hours later, counted and seeded for colony formation. After 10 days, cells were washed with PBS, fixed for 10 minutes in 100% methanol, stained with crystal violet, and colonies were counted. The % survival fraction for a given dose/siRNA was calculated, and dose-response curves were plotted.

### Western blotting analysis

Cell pellets were lysed in RIPA buffer (50 mM Tris pH7.4, 140 mM NaCl, 1% NP40, 0.5% sodium deoxycholate, 0.1% sodium dodecyl sulphate) containing phosphatase and protease inhibitor tablets (Roche). Cell pellets were lysed for 20 minutes on ice, after which nuclear and cellular debris was removed by centrifugation at 13,000 RPM for 10 minutes at 4°C. 10–20 μg of protein lysates were electrophoresed on NuPage precast gels (Invitrogen) and immunoblotted with anti-NF1 (Bethyl Antibodies), anti-phospho-SRC (Tyr416) (Cell Signalling Technology [CST]), anti-SRC (CST), phospho-ERK (CST), phospho-MEK (CST) total ERK (Cell signaling Technology), total MEK (CST), phospho-HER2 (CST), total HER2 (Santa Cruz), phospho-Insulin Receptor/IGF1R (CST), total insulin receptor (CST), phospho-FAK (Tyr925) (CST), total FAK (CST). As loading controls, vinculin (Santa Cruz) and beta-actin (Sigma) were used. Membranes were then incubated in anti-IgG-HRP (CST) and chemiluminescent detection (Luminata Cresendo, Millipore).

### Quantitative real-time PCR

RNA was extracted from cells using STAT-60 Trizol. Chloroform was added and the RNA was extracted from the inorganic phase using isopropanol. cDNA was synthesized by reverse transcription using First strand DNA synthesis kit (Roche), and diluted to 25 ng/ul. 62.5 ng of each cDNA sample was used for each polymerase chain reaction, which was performed in triplicate for each sample. Relative mRNA levels of each gene of interest was quantified using Syber Green (Roche) as a flouresence marker, and analyzed using Light Cycler LC480 (Roche). Melt curve analysis was performed following each PCR run. Beta actin primers were used as a house keeping gene for normalization. Data was analysed using the ddCT method. qPCR primers sequences are defined in Supplementary Information 1.

### Statistical and drug relationship analysis

Data are presented as the mean ± S.E.M from at least three independent experiments. *P* values were calculated using a Student's *t*-test, or in the case of growth sensitivity assays significance was calculated by extra-sum-of squares *F*-test (^*^p < 0.05, ^**^p < 0.01, ^***^p < 0.001) using GraphPad Prism. Quantification of drug synergism and antagonism were conducted using CalcuSyn Software. Combination index (CI value) < 1 indicates drug synergy.

## SUPPLEMENTARY TABLES AND FIGURES





## Abbreviations

MEK: mitogen activated protein kinase kinase
ERK: extracellular signal related kinase
HER2: human epidermal growth factor receptor
CI: combination index
IGF: insulin growth factor
IGF2: insulin-like growth factor 2
IR:A: insulin receptor isoform A
IR:B: insulin receptor isoform B
IGF1R: insulin-like growth factor 1 receptor
IGF2: insulin growth factor 2
EGFR: epidermal growth factor reccptor
EMT: epithelial to mesenchymal transition
MET: mesenchymal to epithelial transition
shRNA: short hairpin RNA
siRNA: small interfering RNA
TSG: tumour suppressor gene
PTTG1: Pituitary transforming gene 1
qPCR: quantitative-polyermase chain reaction
S.E.M: standard error of mean
EOC: epithelial ovarian cancer
MAPK: mitogen activated protein kinase
FAK: focal adhesion kinase
HFF: human foreskin fibroblasts
NF1: neurofibromin 1
RAF: Rapidly Accelerated Fibrosarcoma
