# Supplementary material for: Activation of MAPK signalling results in resistance to saracatinib (AZD0530) in ovarian cancer
Source: Oncotarget. 2017 Dec 20;9(4):4722–36. doi: 10.18632/oncotarget.23524 (PMC5797008; doi:10.18632/oncotarget.23524)
Supplement: Supplementary file 1 [file oncotarget-09-4722-s001.pdf]

Activation of MAPK signalling results in resistance to saracatinib (AZD0530) in ovarian cancer

SUPPLEMENTARY MATERIALS

Cell lines

Human foreskin fibroblast (HFF) cells and TOV112D cells were sourced from the American Type Culture Collection (ATCC). HFF cells were cultured in DMEM media containing 10% FBS. The TOV112D is an endometriod cell cancer cell line [1], and was cultured in RPMI media supplemented with 10% FBS, 1% L-glutamine and 1% sodium pyruvate. IGROV1 is a mixed ovarian cancer cell line [2, 3] and was purchased as part of the NCI60 cell line panel from Charles River Laboratories, and were cultured in were cultured in RPMI media containing 10% FBS and1% l-glutamine. MCF10A WT parental and MCF10A P53–/– cells were purchased from Horizon Discovery and were cultured in DMEM/F-12 including 2.5 mM L-glutamine and 15 mM HEPES, supplemented with 5% horse serum, 10 µg/mL insulin, 20 ng/mL hEGF, 0.5 µg/mL hydrocortisone, 0.1 µg/mL cholera toxin. MDA-MB-436-EV and MDA-MB-436 -BRCA1 (Ms N Buckley, Queens University Belfast, UK) were generated by transfecting the BRCA1-mutant MDA-MB-436 cells with either empty vector pcDNA3.1 or pcDNA3.1-BRCA1, followed by selection in 300 µg/mL G418, and were maintained in 50:50 ratio of RPMI-1640 and Leibovitz's L-15 medium supplemented with 10% fetal calf serum containing 300 µg/ml G418. All cell lines were routinely tested for mycoplasma contamination. Stable shRNA mediated knockdown of SRC in TOV112D cells was performed by lentivirus infection, using the ViraPower™ Lentiviral Expression Systems (Life Technologies). Briefly, HEK293FT cells were transfected with 3 µg of shRNA DNA (Sigma), along 9 µg of ViraPower™ Packaging Mix using Lipofectamine 2000 (Life technologies) according to manufacturers guidelines. 72 hours post transfection, virus particles were collected. To infect TOV112D cells, virus particles were diluted 1:5 in full TOV112D growth medium, and supplemented with 5 µg/ml polybrene (Millipore). 72 hours post infection, media was removed from TOV112D cells and replaced with normal growth media containing 0.5 µg/ml puromycin. SRC knockdown clones were isolated by dilution cloning; once cells reached confluency they were trypsinized and resuspended in 10 mls of growth media and 5µl of the cell suspension was plated into a 15 cm plate. A number of colonies were picked and expanded, and knockdown of SRC was confirmed by western blotting.

siRNA screening

Human foreskin fibroblast cells were reverse transfected with the siRNA library (Qiagen) using Lipofectamine RNAiMax reagent as per manufacturer's instructions (Life Technologies). Cells were transfected with 10 nM siRNA. 24 hours later media was changed to normal growth media containing either DMSO or 10 µM saracatinib, and 72 hours later cell viability was assayed using the Cell Titre Glo assay (Promega). The sensitivity of the screen was monitored by PLK1 siRNA causing a reduction in viability of more than 90%, when compared to transfection with a non-targeting siRNA control (All-stars). The siRNA screen was performed once and the average robust z-scores of the 3 independent siRNAs targeting each gene was determined for each cell line.

Real time qPCR Primers

Insulin receptor isoform A
Forward: GTTTTCGTCCCCAGGCCATC
Reverse: CCAACATCGCCAAGGGACCT

Insulin receptor isoform B
Forward: CACTGGTGCCGAGGACCCTA
Reverse: GACCTGCGTTTCCGAGATGG

HER2/ERBB2
Forward: GGGAAACCTGGAACTCACCT
Reverse: AGCGATGAGCACGTAGCC

NF1
Forward: GCTCACAAAGACACCAAAGTTTC
Reverse: TTTTGTTCGCTCTGCTGAAGT

IGF2
Forward: ACA CCC TCC AGT TCG TCT GT
Reverse: GAA CAC GCA CTC CTC AACGA

siRNA oligonucleotides
NF1-A TACAGTAATSGCACTAACCAA
NF1-B CTCCATCGAATCATCACCAAT
SRC-A CGGCTTGTGGGTGATGTTTGA
SRC-B CTCCATGTGCGTCCATATTTA

HER2 -A AACAAAGAAATCTTAGACGAA
HER2-B CACGTTTGAGTCCATGCCCAA
INSR-ATCGAACGATGTTGGACTCATA
INSR-B CAACGGGAGTCTGATCATCA

REFERENCES

1. Provencher DM, Lounis H, Champoux L, Tétrault M, Manderson E, Wang J, Eydoux P, Savoie R, Tonin P, Mes-Masson A. Characterization of four novel epithelial ovarian cancer cell lines. Vitr Cell Dev Biol - Anim. 2000; 36:357–61. https://doi.org/10.1290/1071-2690(2000)036<0357:COFNEO>2.0.CO;2.
2. Domcke S, Sinha R, Levine DA, Sander C, Schultz N. Evaluating cell lines as tumour models by comparison of genomic profiles. Nat Commun. 2013; 4. https://doi.org/10.1038/ncomms3126.
3. Bénard J, Da Silva J, De Blois MC, Boyer P, Riou G, Duvillard P, Chiric E. Characterization of a Human Ovarian Adenocarcinoma Line, IGROV1, in Tissue Culture and in Nude Mice. Cancer Res. 1985; 45:4970–9.

Supplementary Table 1: siRNA target genes (tumour suppressor genes or genes whose loss has been implicated in cancer development). See Supplementary_Table_1

Supplementary Table 2: List of resistant hits

Gene	Average Z-score	Gene	Average Z-score
DLC1	41.03	TUSC4	2.48
ST7L	35.74	KSR2	2.45
CYLD	19.23	GLTSCR1	2.42
KSR1	14.25	ST14	2.34
TUSC3	12.74	BRCA1	2.19
BRIP1	9.40	HSP90B1	2.19
LATS2	9.39	GLTSCR2	2.18
RAD54L	6.53	BRMS1L	2.12
RB1CC1	6.38	TSG101	2.06
BAP1	5.79	MLH1	2.01
ST13	5.68	LZTS2	1.99
LATS1	5.14	NF1	1.97
Tnfrsf10b	4.85	CAV2	1.90
NBL1	4.44	FBXW7	1.90
GADD45G	4.40	PHB	1.81
CDH19	4.39	CDKN3	1.74
CBX4	3.58	RBBP7	1.72
ATM	3.31	TSSC4	1.71
TAL1	3.26	IFFO1	1.67
FAT1	3.08	MCC	1.65
TP53	3.01	SMAD4	1.61
PTEN	2.84	GADD45B	1.48
TNFRSF10A	2.80	GADD45A	1.39
ST18	2.78	E2F1	1.35
RASSF1	2.76	BAX	1.32
OPCML	2.65	CNKSR1	1.21
PTTG1	2.58		

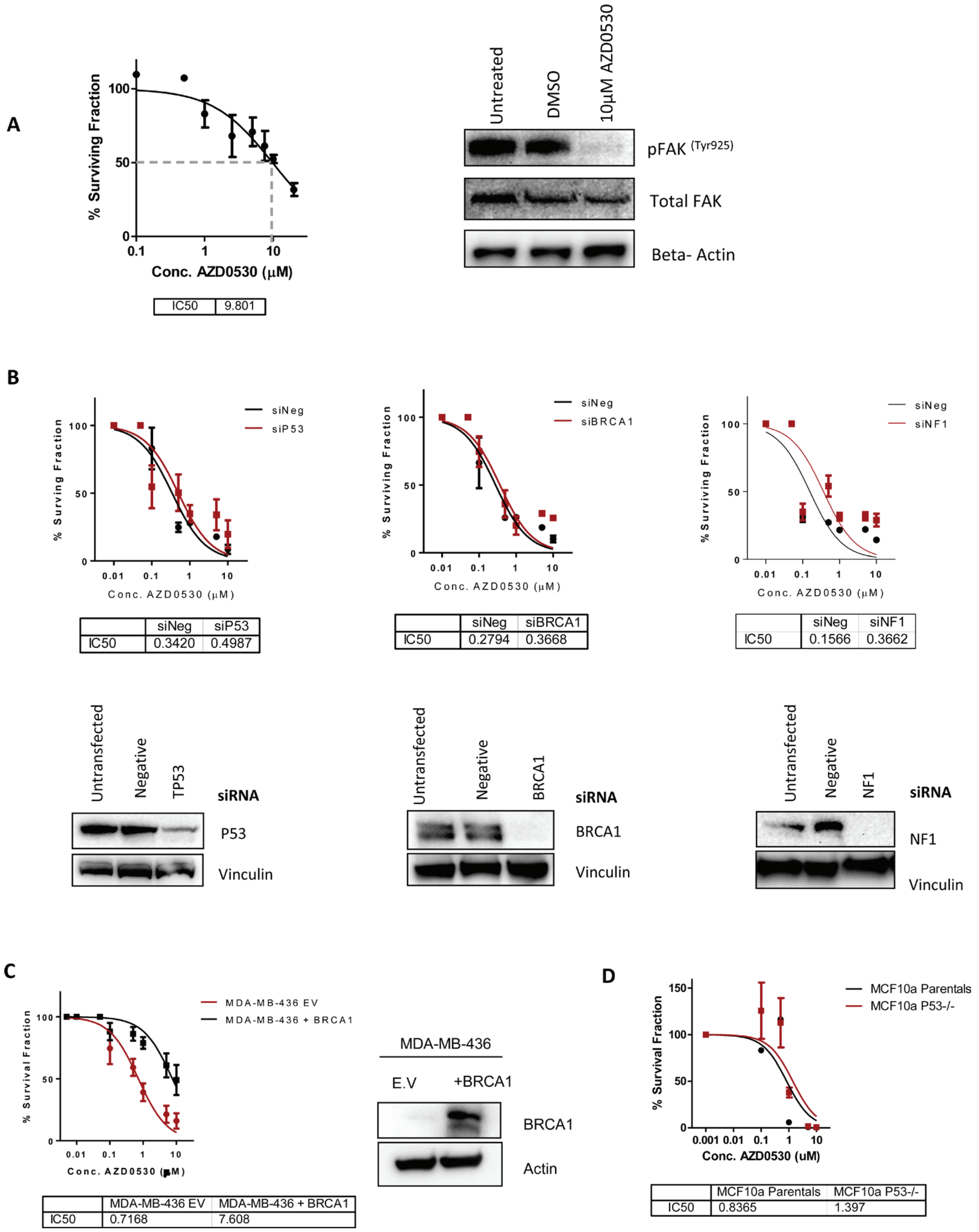

Supplementary Figure 1: Validation of hits identified in siRNA screen. (**A**) Cell viability assay showing sensitivity of human foreskin fibroblast cells following treatment with increasing doses of AZD0530 for 72 hours. Western blot assessing the effect of 10 μM saracatinib (AZD0530) on levels of phosphorylation of FAK (Tyr925). Total FAK and beta-actin are shown as loading controls. (**B**) 10 day cell count assay assessing sensitivity of human foreskin fibroblasts to increasing concentrations of AZD0530 following transfection with All Stars negative control siRNA, P53, BRCA1 or NF1 targeted siRNA. IC50 values were calculated and are shown. Corresponding western blot below showing expression of P53, BRCA1 and NF1 proteins levels following siRNA mediated knockdown in of *TP53, BRCA1* and *TP53* in HFF cells. Vinculin levels are shown as a loading control. (**C**) 10 day colony formation assay showing sensitivity of MDA-MB-436 E.V and MDA-MB-436 + BRCA1 cells to increasing concentrations of AZD0530. IC50 values were calculated and are shown. Corresponding western blot showing expression of BRCA1 in MDA-MB-436 E.V and MDA-MB-436 + BRCA1 cells. Actin levels are shown as a loading control. (**D**) 10 day colony formation assay showing sensitivity of MCF10A parental and MCF10A P53–/– cells to increasing concentrations of AZD0530. IC50 values were calculated and are shown.

A

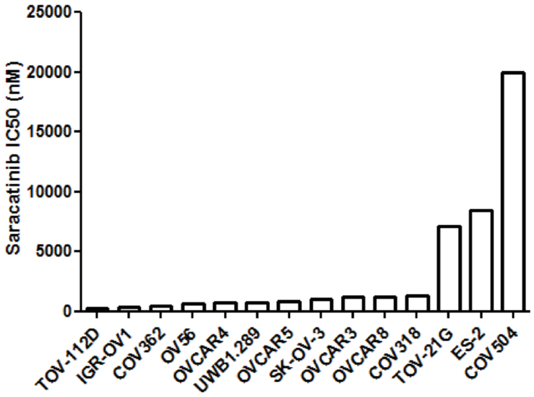

B

Cell line	Saracatinib IC50 (nM)
SUM159	21
TOV-112D	230
IGR-OV1	370
COV362	490
OV56	610
OVCAR4	690
UWB1.289	760
OVCAR5	810
SK-OV-3	1020
OVCAR3	1160
OVCAR8	1170
COV318	1260
TOV-21G	7080
ES-2	8450
COV504	20000

Supplementary Figure 2: Sensitivity of a panel of ovarian cells to saracatinib (AZD0530). (**A**) 10 day colony formation assay assessing sensitivity of 15 ovarian cancer cell lines to AZD0530. IC50 values are shown. (**B**) IC50 values for AZD0530 for each ovarian cell line.

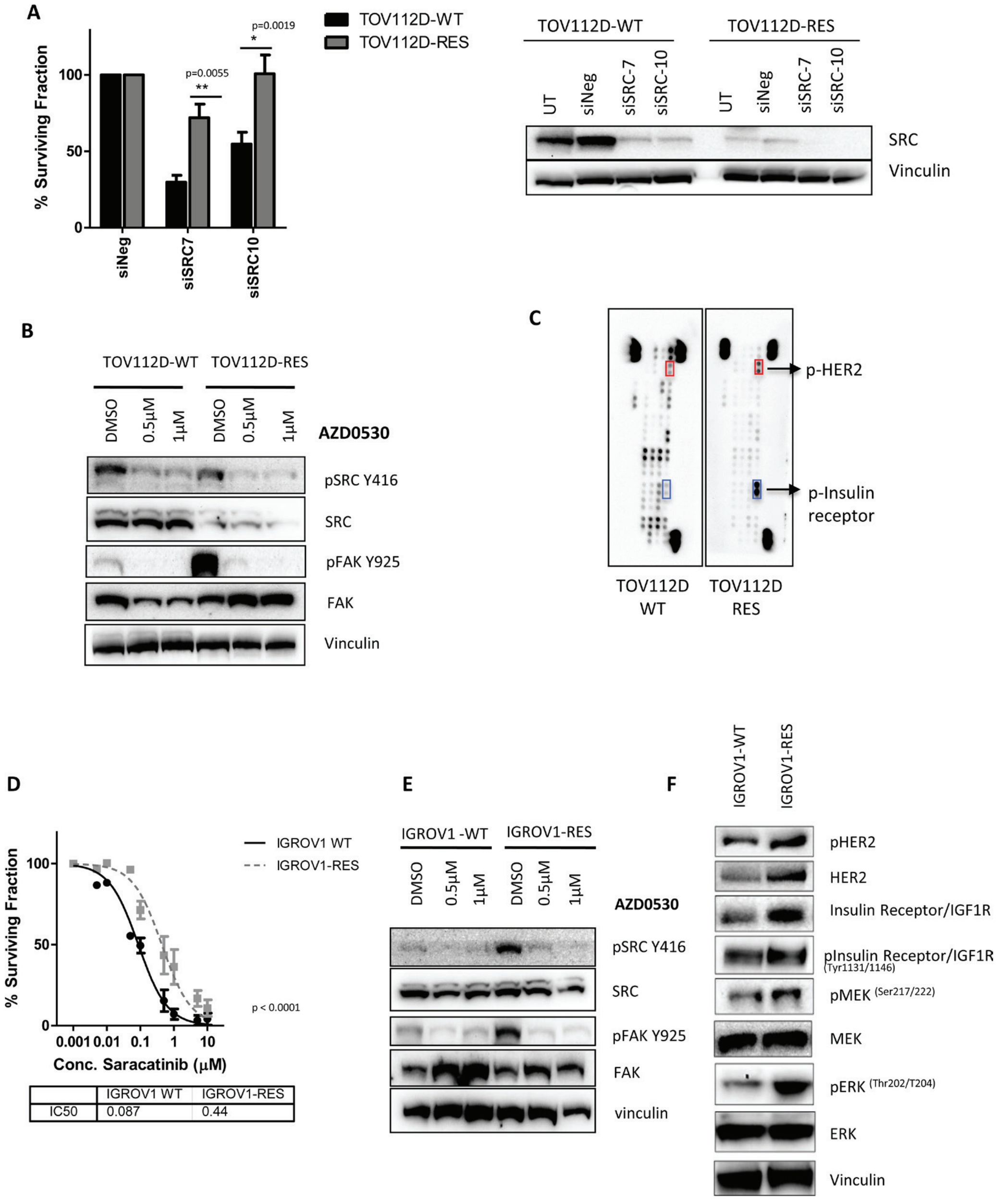

Supplementary Figure 3: Characterisation of saracatinib (AZD0530) resistant ovarian cell lines. (**A**) Colony formation assay assessing sensitivity of TOV112D-WT and TOV112D-RES cells to knockdown of SRC using two independent siRNAs. Corresponding western blot showing levels of SRC expression in TOV112D-WT and TOV112D-RES cells following knockdown of SRC with two independent siRNAs. (**B**) Western blot showing levels of phosphorylated SRC (Tyr416), total SRC, phosphorylated FAK (Tyr925) and total FAK in TOV112D-WT and TOV112D-RES cells following treatment with 0.5 μM and 1 μM AZD0530 for 72 hours. (**C**) Phopsho-RTK array demonstrating phosphorylation of 49 RTKs in TOV112D-WT and TOV112D-RES cells. (**D**) Colony formation assay assessing sensitivity of IGROV1-WT and IGROV1-RES cells to increasing concentrations of saracatinib. (**E**) Western blot showing levels of phosphorylated SRC (Tyr416), total SRC, phosphorylated FAK (Tyr925) and total FAK in IGROV1-WT and IGROV1-RES cells following treatment with 0.5 μM and 1 μM AZD0530 for 72 hours. (**F**) Western blot showing levels of phosphorylated HER2 (Tyr1248), total HER2, phosphorylated insulin receptor/IGF1R (Tyr1131/1146), total insulin receptor, phosphorylated MEK (Ser217/222), total MEK, phosphorylated ERK (Thr202/Tyr204) and total ERK in IGROV1-WT and IGROV1-RES cells. Vinculin levels are shown as a loading control.
